# The Participation of Older Persons in the Adoption of Age‐Friendly Care Models in Hospital Settings: A Scoping Review

**DOI:** 10.1111/nhs.70274

**Published:** 2026-01-04

**Authors:** Nick Anthony Millar, Kathleen F. Hunter, Sherry Dahlke, Kaitlyn Tate, Ian Jefferson Alagadan, Matthias Hoben

**Affiliations:** ^1^ Faculty of Nursing, College of Health Sciences University of Alberta Edmonton Alberta Canada; ^2^ School of Health Policy and Management, Faculty of Health York University Toronto Ontario Canada

**Keywords:** acute care hospital, age‐friendly care, care model, older persons, patient engagement, patient participation, scoping review

## Abstract

Hospitals are increasingly adopting age‐friendly care (AFC) models to address the limitations of traditional care models in the care of older persons. Integrating older persons' participation in this adoption process could lead to hospital care that is tailored to their needs and preferences. However, a comprehensive understanding of older persons' participation in AFC adoption remains limited. Thus, we conducted a scoping review following the JBI methodology and the PRISMA‐ScR reporting guideline to identify and examine the breadth of evidence on this topic. We searched six health science databases in December 2022 (updated in February 2024) for peer‐reviewed research studies describing older persons' participation in the adoption of AFC models in hospital settings. Five unique studies reported in six articles from 2307 screened articles met our inclusion criteria. Older persons' participation appeared primarily at the organizational design and governance level when responding to surveys and interviews, providing verbal feedback (consultation) and, to a lesser extent, joining expert panels as members (involvement). Participation occurred during the implementation phase of AFC adoption. Barriers and facilitators influencing participation included patient‐related and organizational‐related factors. Our review highlighted the limited scope and existing knowledge on older persons' participation in AFC adoption, raising a concern that AFC models may be adopted without adequate consideration of older persons' perceived care needs and preferences.

## Key Points

- Older persons rarely participate in the adoption of AFC models in hospitals, with their participation typically confined to consultation rather than involvement, partnership, and shared leadership.
- Using the NPT, participation of older persons was mainly aligned with the constructs of coherence and reflexive monitoring during the earlier implementation phase of adoption, with no evidence in the embedding and integration phases.
- We identified that further research is needed to improve and promote meaningful participation of older persons in adopting AFC models in acute care hospitals.

## 1 Background

The disconnect between traditional care models and the care needs of hospitalized older persons, resulting in poor care outcomes, has been a longstanding concern in the hospital care of older persons (Loyd et al. 2020; Mudge et al. 2019; Palmer 2018; Zisberg et al. 2015). Traditional care models in hospitals, often rooted in biomedical and paternalistic perspectives, were originally designed to address single, acute, episodic illnesses of younger, independent populations (Arakawa Martins et al. 2021; Huang et al. 2011; Kydd and Fleming 2015). These care models have been proven to be problematic, leading to functional decline and poor care outcomes as they fail to fully address the diverse care needs of hospitalized older persons (Arakawa Martins et al. 2021; Giguere et al. 2018; Parke et al. 2014). Zisberg et al. (2015) reported that 41.2% of hospitalized older persons experienced functional decline during hospitalization, which increased to 46.3% 1 month after discharge from the hospital. Meanwhile, Mudge et al. (2019) estimated that at least 44% of hospitalized older persons suffered from one or two hospital‐associated complications during their hospital stay. Hospital practices, services, care protocols, organizational values, and physical environments in traditional care models typically prioritize curative approaches that rarely preserve, maintain, or improve older persons' functional independence, psychological well‐being, and cognitive ability (Mudge et al. 2019; Zisberg et al. 2015).

The concern arising from the misalignment of traditional care models to the needs of older persons in hospital is further heightened by their intersecting vulnerabilities, including multiple chronic conditions, cognitive and/or physical impairment, and social identities such as gender, ethnicity, sexual orientation, religious or spiritual beliefs, and social class (Nguyen et al. 2021; Rahemi and Williams 2020; Suntai et al. 2024). Existing traditional care models may be limited in their capacity to explore and integrate these complexities in the hospital care of older persons (Ploeg et al. 2017), potentially resulting from their narrow focus and performance‐based priority setting (Giguere et al. 2022). As a result, older persons experience poor care outcomes and hospital‐associated complications, which are linked to prolonged hospital stays, delirium, falls, pressure injuries, immobility, incontinence, functional decline, and admissions to nursing homes (Mudge et al. 2019; Zisberg et al. 2015). Older persons with such complications had a longer length of stay (9.1 days [SD 7.4] vs. 6.8 days [SD 4.1], *p* < 0.001), were more likely to be discharged to a facility (31% vs. 11%, *p* < 0.0001), and had higher six‐month mortality (14% vs. 7%, *p* = 0.02) than older persons without hospital‐associated complications (Mudge et al. 2019). These poor outcomes are alarming, especially since older persons constitute the largest group (approximately 51%) of patients in adult inpatient hospitals (Canadian Institute for Health Information 2025; National Center for Health Statistics 2019).

Age‐friendly care (AFC) models have been proposed as a critical strategy to address the issues described above (Karami et al. 2023). AFC models are multifaceted, person‐centred approaches that involve the physical design of healthcare settings, practice guidelines, culture change, and interdisciplinary care to mitigate the harms and promote positive outcomes of hospital care among older persons (Boltz et al. 2010; Karami et al. 2023; Palmer 2018). Some of the prominent AFC models include the Nurses Improving Care to Health System Elders (NICHE) (Fulmer et al. 2002), the Yale Model of Care for the Elderly (Inouye et al. 1993), the Acute Care for the Elderly (ACE) unit (Palmer 2018), and the 4Ms Age‐Friendly Health system (Fulmer et al. 2018; Pelton et al. 2017). Initial evaluation of AFC models suggested improvements in older person care, including reduced hospital length of stay (Brennan et al. 2019), prevention of functional decline (Squires et al. 2021), decreased mortality rates, improved patient outcomes (Khadaroo et al. 2020), increased patient and healthcare provider satisfaction (Arain et al. 2020), and improved cost containment (Hofmeister et al. 2020). However, studies often have methodological limitations, leading to heterogeneous findings, and the adoption of AFC models remains sporadic, slow, and small‐scale (Allen et al. 2020; Rogers et al. 2022).

Additionally, studies reporting the development, implementation, and evaluation of AFC models rarely mention older persons' participation (Inouye et al. 1993; Palmer 2018; Parke and Stevenson 1999; Pelton et al. 2017; Ryan et al. 2022; Sinha et al. 2018). The lack of involvement of older persons in adopting AFC models suggests that many of these models are primarily developed from the perspectives of healthcare professionals, organizations, and researchers. This is of concern, as it has been suggested that patient participation in healthcare improvement ensures that proposed changes and innovations are person‐centred and aligned with their needs and preferences (Baker et al. 2016; McNeil et al. 2022). Allowing patients to participate may also help address potential issues in the adoption process, such as tailoring healthcare improvements to local contexts (Bergerum et al. 2019). These benefits highlight the importance of older persons' participation in adopting AFC models as a critical component to promoting person‐centred care and aligning these models to their perceived needs (Boltz et al. 2010). Unfortunately, existing literature suggests that older persons are rarely involved in healthcare improvements in hospitals (Liang et al. 2018; Modigh et al. 2021), potentially explaining the limited adoption of AFC models. From a pragmatic standpoint, older persons' participation may increase the AFC models' adaptation to local contexts, values, previous experiences, and needs (Baker et al. 2016) and facilitate a smooth adoption process that could address the current challenges when adopting AFC models. However, the knowledge about the nature and breadth of evidence on older persons' participation in adopting AFC models remains missing in the literature.

## 2 Aim and Review Question

To the best of our knowledge, no review has summarized the evidence on the participation of older persons in adopting AFC models in acute care hospitals. By conducting this scoping review, we aimed to identify and examine the available evidence on older persons' participation in AFC adoption and gaps for future research on this topic. Our review question was “What is the nature and extent of older persons' (population) participation in adopting AFC models (concept) in acute care hospital settings (context)?”

## 3 Methods

### 3.1 Review Design

Our scoping review followed the Joanna Briggs Institute (JBI) methodology (Peters, Godfrey, et al. 2020; Peters, Marnie, et al. 2020) and the PRISMA Extension for Scoping Reviews (PRISMA‐ScR) recommendations (Tricco et al. 2018) to ensure methodological rigor in conducting our review (File S1). The Patient and Family Engagement Framework (PFEF) and Normalization Process Theory (NPT) guided the directed content analysis of the extracted data from the included studies. We used the PFEF to categorize the levels and degrees of participation, as well as the barriers and facilitators influencing older persons' participation in adopting AFC models. In parallel, we used the three phases and the four constructs of the NPT to map when and how older persons participated in the AFC model adoption process. Our review protocol was registered with the Open Science Framework (https://doi.org/10.17605/OSF.IO/KM836).

### 3.2 Theoretical Positioning

#### 3.2.1 Patient and Family Engagement Framework

We recognized that the terms participation and engagement were frequently used interchangeably in the literature. To distinguish between terms, we defined participation as the activities that patients perform to improve their care and health services (Jerofke‐Owen et al. 2023). Meanwhile, engagement was defined as the actions and efforts of healthcare providers and health organizations to motivate and empower patients to take ownership of their care and contribute to health service design (Jerofke‐Owen et al. 2023). For this scoping review, we used the term participation to describe the older persons' actions in organizational efforts to adopt AFC models and categorized participation using the PFEF.

**FIGURE 1:**
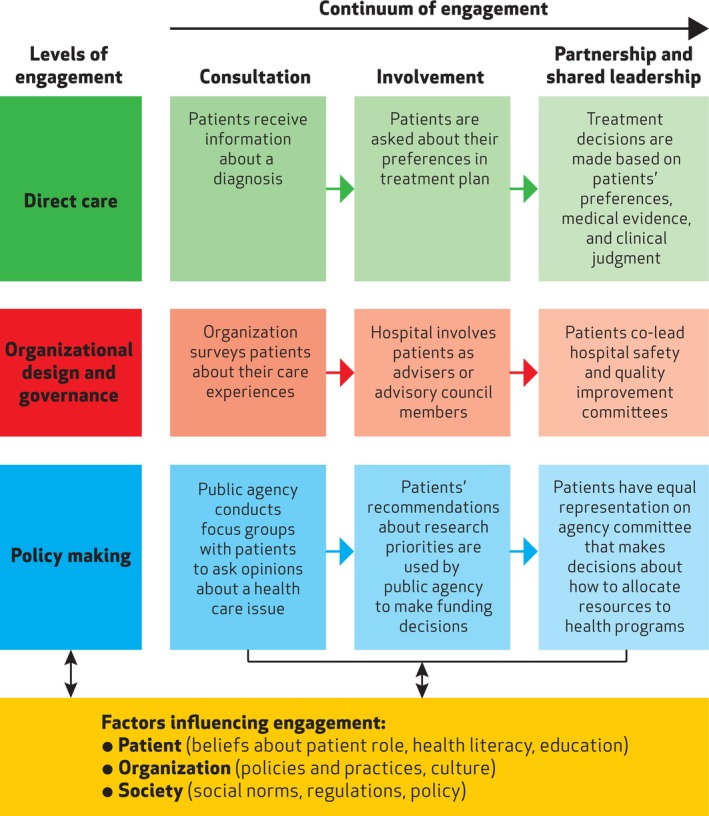
Patient and family engagement framework (Carman et al. 2013) reprinted with permission.

Based on the PFEF (Figure 1), participation can occur on three levels: direct care, organizational design and governance, and policy‐making (Carman et al. 2013). At each level, participation can take place through consultation, involvement, or partnership and shared leadership (describing a continuum of low to high engagement) (Carman et al. 2013). Various patient, organization, and society‐level factors influence the degree and level of participation that will occur (Carman et al. 2013).

### 3.3 Normalization Process Theory

**FIGURE 2:**
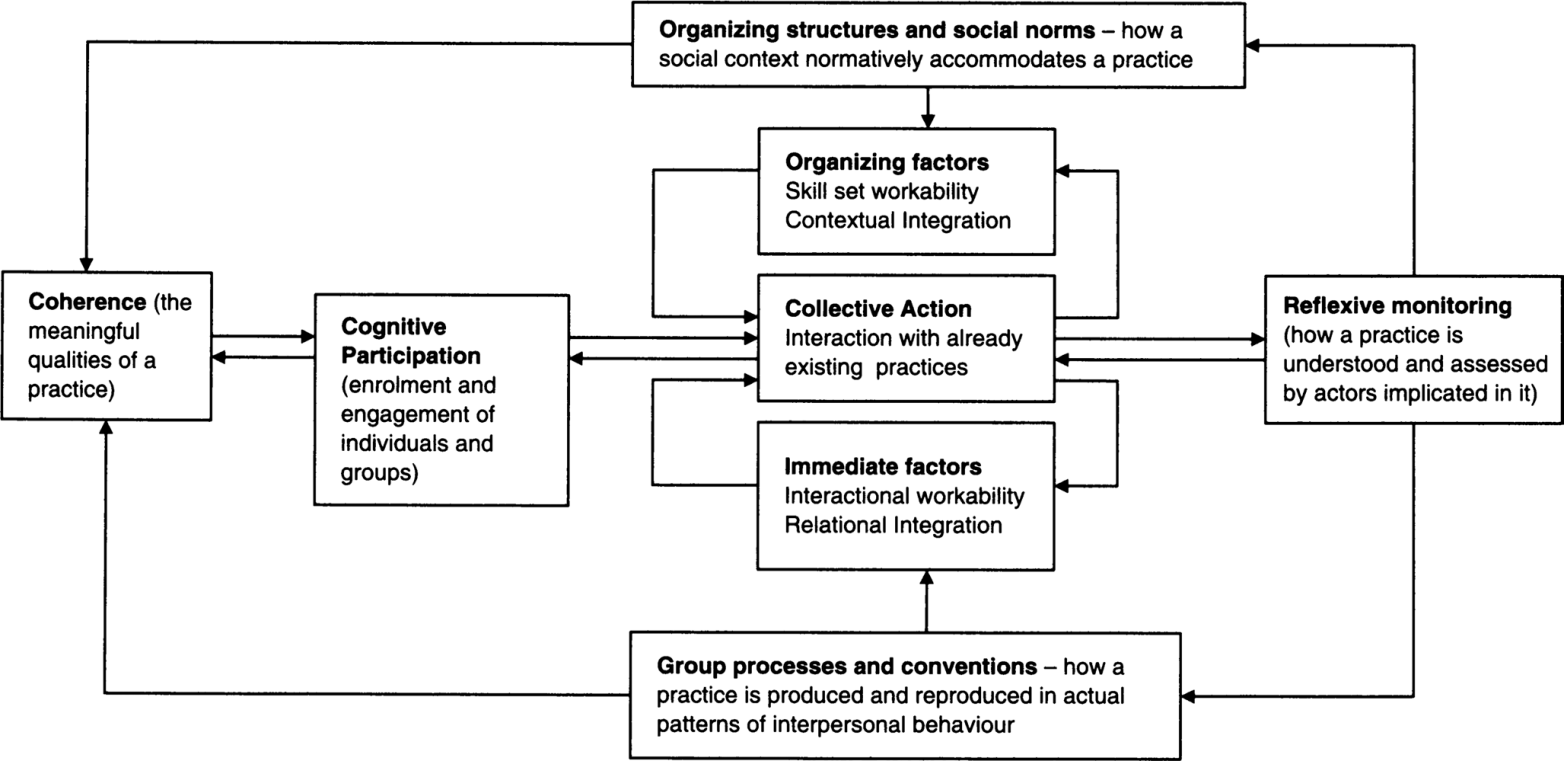
Model of the components of normalization process theory (May and Finch 2009) reprinted with permission.

The NPT is a middle‐range theory that explains how and why new complex healthcare innovations, such as introducing AFC models, are normalized or adopted as standard practice in an organization (Figure 2) (Finch et al. 2012). Its application in quality improvement and change management in healthcare is well‐documented in the literature, particularly when interventions are tailored to the local context and are sustained in practice (May et al. 2018).

Normalization is achieved through the three phases of implementation, embedding, and integration (May and Finch 2009). Implementation pertains to how an organization introduces new practices or technology into action (May et al. 2009). Embedding relates to incorporating new practices or interventions into the daily work of individuals (May et al. 2009). Lastly, integration involves replicating and sustaining a new practice, technology, or healthcare intervention across the organization (May et al. 2009). In keeping with the NPT, we described the normalization of AFC models as the adoption of AFC models that has three phases of implementation, embedding, and integration.

The NPT has four constructs: (a) coherence or the initial work of defining and being familiar with the new practice, technology, or healthcare intervention; (b) cognitive participation or gaining an understanding of the process that organizations and healthcare professionals undergo to implement the new practice, technology, or healthcare intervention; (c) collective action or the work involved to execute the new practice, technology, or healthcare intervention; and (d) reflexive monitoring or the continuous formal and informal evaluation while the new practice, technology; or healthcare intervention is in place (May and Finch 2009). In this scoping review, we used these four constructs to categorize how older persons participate in the adoption of AFC models.

### 3.4 Eligibility Criteria

We included published, peer‐reviewed research studies describing the participation of older persons in adopting the AFC model in hospitals. Older persons were defined as individuals aged 65 years and older. We excluded gray literature during the screening stage to maintain our focus on identifying only peer‐reviewed research and to assess the current state of research in this area. References solely focusing on AFC models in other settings (e.g., community care, long‐term care, primary care) were excluded. Our complete inclusion and exclusion criteria are summarized in Table 1.

**TABLE 1:** Eligibility criteria for scoping review.

	Inclusion criteria	Exclusion criteria
Study focus	Studies that describe the adoption of AFC models in hospital settings	Studies that describe AFC models for settings other than hospitals
Study design	Quantitative research Qualitative research Mixed‐method research	Commentaries, editorials, opinion papers, discussion papers, or case reports Literature reviews (but we considered research studies included in available literature reviews for inclusion) Research protocols Quality improvement reports Abstracts and conference reports
Types of evidence	Published peer‐reviewed original research	Gray literature (published outside peer‐reviewed journals)
Study setting	Acute care hospitals including specific acute care areas such as surgical units, intensive care units, emergency, and acute medicine units Healthcare settings in general, as long as hospital settings were included, and relevant results are reported for hospital settings separately	Long‐term care settings or nursing homes Non‐acute care settings such as complex continuing care or inpatient rehabilitation units Day hospital programs Outpatient clinics
Study population	Older persons aged 65 years and older	Patient population of 64 years and younger
Study outcome	Participation of older persons in the adoption process of AFC models	Older Persons' participation is missing in the adoption of AFC models

### 3.5 Search Strategy

The literature search consisted of three steps following the JBI scoping review guideline: (a) an initial search in two databases to identify search terms, (b) a search of all databases for citations, and (c) a manual search of the reference list of included studies (Peters, Godfrey, et al. 2020). We developed our search protocol (File S2) in consultation with a health science librarian (Peters et al. 2022) using the search terms identified during the initial search of CINAHL and Medline databases. Our search protocol consisted of key terms, search terms, and index terms for AFC models, older persons, and acute care settings combined with the Boolean operator “and”. The EBSCO and OVID platform search filters for geriatrics from the University of Alberta Library (Campbell 2021a, 2021b) were used for “older persons”. We conducted a comprehensive search of CINAHL, MedLine, Global Health, PsychInfo, Scopus, and Embase databases using the search protocol in December 2022 and repeated in February 2024. Lastly, two independent reviewers (NM and IA) completed a manual bibliographic search of the reference list of the included studies to identify any additional relevant articles missed in the database search.

### 3.6 Study and Source of Evidence Selection

Potentially relevant citations from the database searches were uploaded to Covidence (https://www.covidence.org/), a systematic review software. Two reviewers (NM and IA) initially screened 25 randomly selected titles and abstracts, achieving a 96% agreement that met the recommended threshold of 75% agreement (Peters, Godfrey, et al. 2020). Subsequently, NM and IA each completed the title and abstract, and full‐text screening stages independently, with KH and SD as third reviewers to resolve disagreements (Peters, Marnie, et al. 2020). After the title and abstract screening, we obtained the full text of all studies, read in completion, and screened independently by two reviewers for inclusion. One study in the full‐text review stage was published in Italian and was translated into English using DeepL Pro (DeepL SE 2018).

### 3.7 Data Extraction

NM and IA extracted the data from included studies using a data extraction tool created by the research team in COVIDENCE. The same reviewers performed a pilot of the data extraction tool using one included article, selected randomly, to ensure consistency. Data extracted from the studies included: (1) study characteristics (year of publication, country of origin, author affiliation, and research question, purpose, or aim); (2) study design (setting, sample and sampling methods, data collection methods, involved party group if applicable); (3) summary of AFC models and their components; (4) older persons' participation based on the PFEF; (5) phase and construct of NPT when the participation of older persons occurs in the AFC model adoption process; and (6) barriers and facilitators of older persons' participation using factors identified in the PFEF. The extracted data were exported to a Microsoft Excel sheet, which was reviewed by all authors.

### 3.8 Analysis and Presentation

NM conducted the analysis of the extracted data using descriptive statistics and directed content analysis, and all authors verified all the results. The study characteristics were charted in the summary of the literature table (Table 2) and analyzed using descriptive statistics using the Microsoft Excel program. The AFC models and their components in the included studies were compared and summarized (Table 3).

**TABLE 2:** Summary of included studies.

Study	Glasson et al. (2006)	Kelley et al. (2011)	McComb et al. (2018)	Ryan et al. (2022)	Publication 1: Mudge et al. (2022)
					Publication 2: Mudge et al. (2023)
Country of publication	Australia	Canada	Canada	Canada	Australia
Study aim	To improve the quality of nursing care for older acutely ill hospitalized medical patients through developing, implementing and evaluating a new model of care	To assess the environment of an emergency department (ED) and its impact on care of adults aged 75 and over, using a ‘senior‐friendly’ conceptual framework that included the physical environment, social climate, hospital policies and procedures, and wider health care system	To create an independently led post‐surgical reconditioning program and pilot its implementation while assessing the feasibility and safety of the program	To conduct a scoping review of the literature on system‐based approaches to improving the care of older people in both hospital and community‐based settings and to use the findings to create a broader Senior Friendly Care Framework to guide intersectoral system improvements	Publication 1: To implement and evaluate a ward‐based improvement program (Eat Walk Engage) to more consistently deliver age‐friendly principles of care to older individuals in acute inpatient wards. Publication 2: The objective of this process evaluation was to understand how Eat Walk Engage worked across trial sites.
Design	Convergent Mixed Method Approach, Phase 2 of a Participatory Action Research	Focused Ethnography	Pretest‐Posttest Quasi‐Experimental Study	Scoping Review and Modified Delphi Study	Cluster Randomized Control Trial Prospective Multi‐method Implementation Evaluation
Setting	Acute Medicine Unit	Emergency Department	Surgical Inpatient Unit	Virtual: Expert panel members representing hospitals and community sectors across rural and urban areas in Ontario and one member from Quebec.	Four Medicine and Surgical Inpatient Units across Four Hospitals
Participants	*Premodel Group* 41 older persons 14 nurses *Model Group* 60 older persons 13 nurses	*ED Interviews* 56 older persons Nine (9) proxy decision‐makers *Post Discharge Interviews* 11 older persons Four (4) proxy decision‐makers *Staff Interviews* 61 staff members *Key Informant Interviews* Eight (8) informants from six (6) community agencies *Staff Survey* 26 staff members	*Development Phase of Intervention*: Occupational therapist (*n* = unknown) Physical therapist (*n* = unknown) Older Persons (*n* = unknown) *Pilot Study Phase* 33 older persons in the control group 33 older persons in the intervention group	*Modified Delphi Study Phase* 30 participants that include health administrators, leaders, front‐line clinicians, researchers, policymakers, older adults, and caregivers *Involved Party Consultation* 34 involved parties that include health administrators, leaders, front‐line clinicians, researchers, policymakers, older persons, and caregivers	*Randomized Controlled Trial* Intervention Group 265 Control 274 *Patient Interviews* Pre‐intervention: 42 older persons Post‐intervention: 38 older persons
Reasons for involving older persons	To identify the meaning of quality of nursing care to older persons	The growing population of older persons and high utilization of healthcare services.	Older persons are vulnerable and at risk for adverse events after surgery.	To have an intersectoral approach in defining the Senior Friendly Care Framework	To identify the focus of improvement and strategies and inform about the context.
Findings	Older persons had improvement in satisfaction with care, knowledge about medication, and functional ability. Qualitative findings revealed that nurses were motivated and willing to collaborate to improve the care of older persons but faced barriers such as time constraints, low staffing levels, and lack of confidence in making a change in practice.	ED staff faced difficulties communicating with patients, attending to the older persons' physical, social, and emotional needs, involving family members in care, and promoting teamwork. These findings assisted in formulating recommendations for short‐term and long‐term improvement work based on the Senior Friendly Conceptual Framework.	Their findings indicated that older persons in the intervention group could perform the exercises without adverse events by postoperative day two and showed better Sit‐to‐Stand and Time Up and Go (TUG) scores.	They emphasized the importance of an integrated network between acute care and community services in delivering good care for older persons. After recruiting involved parties from various sectors involving external agencies, older persons, and family caregivers, they redefined the Senior Friendly Care Framework, identifying the 31 defining statements and 10 guiding principles.	Publication 1: The Eat Walk Engage program in four intervention wards resulted in a decline in the incidence of delirium but did not significantly affect other hospital‐associated complications, readmissions, discharge to long‐term care, or mortality. Publication 2: They highlighted the role of site facilitators in identifying different contexts such as leadership, team communication, culture of innovation, patient care culture, and infrastructure or resources and assessing their impact on implementation. Site facilitators were instrumental in conducting ward observations, intervention audits, and patient interviews, which helped tailor the interventions to fit the specific contexts of each ward.

**TABLE 3:** Summary of older persons' participation in adopting AFC models.

Study	Age‐friendly care model and components	Older person participation	Activities completed by older persons	Patient and family engagement framework		Normalization process theory		Barriers	Facilitators
				Degree	Level	Phase	Construct		
Glasson et al. (2006) Australia	New model of nursing care – unnamed Clinical Practice	Identifying Clinical Practice Focus	Responded to a survey to identify priority needs	Consultation	Organizational Design and Governance	Implementation	Coherence	*Patient Factors* Treatment Clinical stability Energy level Cognitive capacity	*Organizational Factors* Assistance with completing surveys
		Model Evaluation	Responded to a satisfaction survey to evaluate a new care model	Consultation	Organizational Design and Governance	Implementation	Reflexive monitoring		
Kelley et al. (2011) Canada	Senior Friendly Care Conceptual Framework Physical Environment Social Climate Hospital Policies and Procedures Healthcare System	Assessment of the Senior Friendliness of Clinical Area	Participated in interviews during their stay in the ED	Consultation	Organizational Design and Governance	Implementation	Coherence	*Patient Factors* Clinical stability Cognitive capacity	*Patient Factors* Family caregivers as proxies
			Participated in interviews after their stay in the ED	Consultation	Organizational Design and Governance	Implementation	Coherence		
McComb et al. (2018) Canada	Elder‐Friendly Approaches to the Surgical Environment – Bedside reconditioning for Functional ImprovemenTs (EASE‐BE FIT) Clinical Practice	Designing the Interventions	Provided feedback to visual instructional materials and logbooks	Consultation	Organizational Design and Governance	Implementation	Coherence	*Patient‐Related Factors* Clinical stability Sensory impairment *Organizational‐Related Factors* Time constraints Misunderstanding the instructions	*Organizational‐Related Factors* Teaching and demonstration by staff Simplicity of Interventions Availability of exercise instructional material and logbooks
		Model Evaluation (Pilot stage)	Provided written feedback on the program using logbooks	Consultation	Organizational Design and Governance	Implementation	Reflexive monitoring		
Ryan et al. (2022) Canada	Senior Friendly Care Framework Organizational Support Process of Care Emotional and Behavioral Environment Ethics in Clinical Care and Research Physical Environment 31 Defining Statements Seven Guiding Principles	Determining AFC model components	Participated as a member of the expert panel	Involvement	Organizational Design and Governance	Implementation	Coherence	None reported	*Organizational‐Related Factors* Introductory webinar sessions for participants Virtual meetings Online feedback
			Provided feedback to the involved party consultation	Consultation	Organizational Design and Governance	Implementation	Coherence		
Mudge et al. (2022), Mudge et al. (2023) Australia	Eat Walk Engage Program Clinical Practice Multidisciplinary Approach Site Facilitators	Identifying Clinical Practice Focus	Participated in the interview to provide feedback on the model before the implementation	Consultation	Organizational Design and Governance	Implementation	Coherence	*Patient‐Related Factors* Clinical stability Cognitive capacity	*Organizational‐Related Factors* Presence of trained site facilitators
		Assessment of Context							
		Model Evaluation	Participated in the interview to provide feedback about the model after implementation	Consultation	Organizational Design and Governance	Implementation	Reflexive monitoring		

To categorize the nature and extent of participation of older persons in the implementation, embedding, and integration of AFC models, we used the PFEF and NPT as the theoretical guides for our directed content analysis (Hsieh and Shannon 2005). Before the analysis, all authors reviewed the definitions, descriptions, and assumptions of the PFEF and NPT, including all of their constructs (Assarroudi et al. 2018). We created a coding matrix (Table 3) using categories derived from the PFEF and NPT, such as level and degree of engagement, influencing factors of engagement, phases of normalization, and normalization constructs (Assarroudi et al. 2018). This coding matrix was used to examine and categorize the extracted data (Table 3).

## 4 Results

### 4.1 Study Selection and Characteristics

The search results and the study inclusion process are presented in a PRISMA diagram (Table 4) (Tricco et al. 2018). Database (*n* = 3269) and manual reference list (*n* = 28) searches identified 3297 articles. After removing 990 duplicates, we screened 2307 titles and abstracts, of which 53 articles were potentially relevant and moved to full‐text screening. Of these articles, six met our inclusion criteria. Two of the included articles reported different aspects of the evaluation of a single program, which we treated as a single study (Mudge et al. 2022, 2023) for a total of five included studies. Details of the included studies are summarized in Table 2. Of the five included studies, one study used a convergent mixed methods design (Glasson et al. 2006), one used a focused ethnography (Kelley et al. 2011), one used a pretest‐post‐test quasi‐experimental design (McComb et al. 2018), one combined a scoping review with a modified Delphi approach (Ryan et al. 2022), and one used a cluster randomized experimental design (Mudge et al. 2022, 2023).

**TABLE 4:** PRISMA‐Scr diagram.

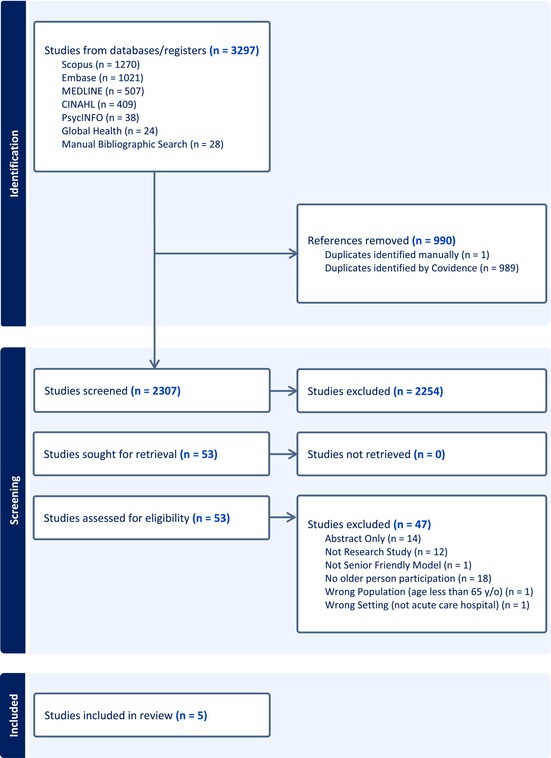

Included studies were published from 2006 to 2023, with three from Canada (Kelley et al. 2011; McComb et al. 2018; Ryan et al. 2022), and two from Australia (Glasson et al. 2006; Mudge et al. 2022, 2023). Glasson et al. (2006) investigated the development and adoption of a new model of care for acutely ill hospitalized older persons that improved their satisfaction with care, knowledge about medication, and functional ability. Kelley et al. (2011) explored the alignment of the emergency department (ED) with a specific AFC model that highlighted the challenges in providing care to older persons in a fast‐paced and chaotic clinical environment. McComb et al. (2018) tested the feasibility and safety of the Elder‐Friendly Approaches to the Surgical Environment—Bedside reconditioning for Functional Improvements (EASE‐BE FIT), a standardized mobility program for postoperative older persons after abdominal surgery. Mudge et al. (2022) and Mudge et al. (2023) published two separate reports on the implementation of the Eat Walk Engage program, a multidisciplinary approach to delivering AFC by addressing the nutrition, hydration, mobility, cognition, and socialization needs of hospitalized older persons.

### 4.2 Main Findings

Findings were grouped into four main categories: AFC models and components, the nature and extent of older persons' participation, the normalization phase, and barriers and facilitators to the adoption of AFC models (Table 3). We identified five AFC models described across the included studies. The participation of older persons consisted of 10 activities that included responding to surveys and interviews, providing verbal and written feedback on interventions, and participating as involved party members in meetings. Of these 10 activities, nine were mainly consultations at the organizational design and governance level of the PFEF. Older persons' participation was limited to the implementation phase and confined to coherence and reflexive monitoring constructs of the NPT.

### 4.3 AFC Models and Components

Included studies described five different AFC models: (1) unnamed new model of care (Glasson et al. 2006), (2) the Senior Friendly Conceptual Framework (Kelley et al. 2011), (3) the EASE‐BE FIT (McComb et al. 2018), (4) the Senior Friendly Care Framework (Ryan et al. 2022), and (5) the Eat Walk Engage Program (Mudge et al. 2022, 2023).

We classified the AFC models as singular or multifaceted based on the number of components (Table 3). Models with singular components mainly focused on specific clinical practice components related to older persons' care. For example, Glasson et al. (2006) developed a new care model that addressed medication regimen knowledge and the functional ability of older persons in hospitals. Similarly, McComb et al. (2018) described the EASE‐BE Fit, a reconditioning program performed by older persons after abdominal surgery. AFC models with multifaceted components consisted of components in addition to clinical practices, such as physical environment (Kelley et al. 2011; Ryan et al. 2022), psychosocial and behavioral environment (Kelley et al. 2011; Ryan et al. 2022), organizational support and structure (Kelley et al. 2011; Mudge et al. 2022, 2023; Ryan et al. 2022), health system (Kelley et al. 2011), and ethical guideline (Ryan et al. 2022) in addition to the clinical practice components.

The physical environment pertained to the hospital's structural and design aspects, such as equipment, lighting, signage, and furniture arrangements that impacted older persons' safety, comfort, functional abilities, emotional well‐being, and cognitive function (Kelley et al. 2011; Ryan et al. 2022). The emotional and psychological environments were the result of interactions between physicians, staff, administrators, older persons, and family caregivers (Kelley et al. 2011; Ryan et al. 2022). This component often influenced the patterns of communication and responsiveness of staff to older persons' emotional and physical needs and to the family caregivers' involvement when caring for older persons (Kelley et al. 2011; Ryan et al. 2022). Organizational support and structure focused on the influence of administrative or leadership structures and functions when providing older person care, including policies, procedures, and guidelines governing care provision and resource allocation (Kelley et al. 2011; Mudge et al. 2022, 2023; Ryan et al. 2022). The health system component was described as the relationship between the acute care sectors and community services in delivering healthcare for older persons (Kelley et al. 2011; Ryan et al. 2022). Ryan et al. (2022) did not specifically identify this as a component, but they described integration and continuity of care, as well as collaboration with organizational partners, in their AFC model's defining statements. The ethical guideline component, identified only in the AFC model by Ryan et al. (2022), highlighted the protection of the rights of older persons in care and in research that included autonomy, choice, dignity, and protection from abuse.

### 4.4 Nature and Extent of Participation

We identified 10 activities older persons perform when participating in adopting AFC models (Table 3). These activities were grouped into categories using the category matrix derived from the theoretical models guiding our scoping review: the PFEF for the level and degree of participation and the NPT for the phase and constructs of when and how the participation occurs in the adoption process of AFC models.

Older persons participated in adopting AFC models through consultation and, to a lesser extent, involvement at the organizational design and governance level in all included studies. Participation by consultation in organizational design and governance occurred when identifying clinical practice focus, developing, evaluating (Glasson et al. 2006; McComb et al. 2018; Mudge et al. 2022, 2023), and redefining (Ryan et al. 2022) AFC models, and in appraising the alignment of the ED to an AFC model (Kelley et al. 2011). Older persons completed surveys (Glasson et al. 2006), participated in interviews (Kelley et al. 2011; Mudge et al. 2022, 2023), and provided feedback on their care or model components (Glasson et al. 2006; McComb et al. 2018; Ryan et al. 2022). Participation through involvement in organizational design and governance occurred when older persons were recruited as members of an intersectoral expert panel of healthcare leaders, clinicians, researchers, policymakers, older persons, and family caregivers for a modified Delphi study that redefined an AFC model (Ryan et al. 2022). None of the studies included older persons' participation in other levels (direct care and policy making) and degree (partnership and shared leadership).

Older persons' participation was mainly determined by researchers and enacted differently in the included studies. Glasson et al. (2006) used the results of their previous study to identify the aspects of care that were important for older persons but were frequently unmet in hospitals (Chang et al. 2003; Hancock et al. 2003). Based on these priorities, nurses selected medication practice and functional ability as the focus of their new care model (Glasson et al. 2006). After implementing the new care model, older persons completed satisfaction surveys to evaluate the new care model (Glasson et al. 2006). Kelley et al. (2011) had older persons participate in interviews during their stay in the ED and after discharge to determine the unit's alignment with their Senior Friendly Conceptual Framework. In the EASE‐Be Fit program, older persons provided feedback on the visual instructional materials and patient logbooks before the trial and about the program after the trial (McComb et al. 2018). Ryan et al. (2022) recruited older persons as members of an expert panel and as part of involved party groups, which helped identify the defining statements and principles of their AFC model. Lastly, older persons were interviewed before and after implementing the Eat Walk Engage Program to ensure the alignment of the interventions with the needs of hospitalized older persons and the contexts of each intervention ward (Mudge et al. 2022, 2023).

### 4.5 Phases and Constructs of Normalization

All included studies incorporated older person participation solely within the implementation phase of the NPT (Table 3). Participation of older persons in the implementation phase of AFC models consisted of identifying clinical practice focus (Glasson et al. 2006; Mudge et al. 2022, 2023), designing the interventions (McComb et al. 2018), determining model components (Ryan et al. 2022), assessment of clinical context (Mudge et al. 2022, 2023), assessment of age‐friendliness of the ED (Kelley et al. 2011), and model evaluation (Glasson et al. 2006; McComb et al. 2018; Mudge et al. 2022, 2023). The included studies mainly reported on the implementation phase and did not describe the embedding or integration phases of AFC model adoption. For this reason, we could not assess the participation of older persons beyond the implementation phase.

Older persons' participation focused on the coherence and reflexive monitoring constructs of NPT. In terms of coherence, older persons contributed to the adoption of AFC models by identifying the problem or the proposed solution (Glasson et al. 2006; Kelley et al. 2011; McComb et al. 2018; Mudge et al. 2022, 2023). Older persons shared their opinions as an expert panel and involved party members (Ryan et al. 2022). Regarding reflexive monitoring, older persons contributed to appraising the impact of the AFC model when they completed surveys (Glasson et al. 2006), participated in interviews (Mudge et al. 2022, 2023), and wrote their feedback in logbooks to evaluate the implementation of AFC models (McComb et al. 2018). None of the participation of older persons corresponded to cognitive participation (activities to improve the understanding of the process of AFC model adoption) or collective action (the performance of activities involved in adopting AFC models) of the NPT.

### 4.6 Barriers and Facilitators to Participation

The barriers and facilitators to older persons' participation identified included patient‐related and organizational‐related factors (Table 3). None of the studies reported any society‐related factors that might influence the participation of older persons.

### 4.7 Barriers

Patient‐related factors were linked to the physiological status of older persons. For example, clinical stability, sensory deficits, energy levels, and cognitive capacity influenced the ability to engage in the adoption process actively. Most barriers were mainly identified by the authors as exclusion criteria with limited explanations of how these criteria might deter older persons from participating. Glasson et al. (2006) reported that older persons declined to participate in the study because they were undergoing procedures, were too ill, or were too tired. Older persons with a mini‐mental state examination (MMSE) score of less than 19 were also excluded from participating in one study (Glasson et al. 2006). Similarly, Kelley et al. (2011) identified clinical instability and cognitive impairment as reasons to exclude older persons. Some older persons declined to participate in performing exercises, citing that it was too soon after the surgery for these exercises (McComb et al. 2018). Poor instructions and time constraints were identified as organizational‐related barriers. McComb et al. (2018) reported that some older persons might have misunderstood the instructions on how to use logbooks and missed documenting their exercises. They also suggested that time constraints may have affected older persons who could not record their adherence to the reconditioning program (McComb et al. 2018).

### 4.8 Facilitators

Facilitators consisted of one patient‐related factor and predominantly organizational factors. The presence of family members was the single patient‐related factor identified as a facilitator in our review. In an attempt to consider the experience of older persons with cognitive impairment or clinical instability, Kelley et al. (2011) interviewed family caregivers as proxies to elicit the experience of older persons' care in the ED. Organizational factors included assistance from dedicated personnel, offering information sessions for older persons, the availability of patient materials, conducting virtual meetings, and the simplicity of intervention design. Glasson et al. (2006) ensured that there were personnel who could assist older persons in completing the satisfaction surveys if required. Similarly, Mudge et al. (2022) and Mudge et al. (2023) trained and employed site facilitators to implement the Eat Walk Engage program and conduct pre‐implementation and post‐implementation patient interviews (Mudge et al. 2022, 2023). McComb et al. (2018) intentionally chose simple exercises that older persons could easily perform and understand. They also implemented the use of logbooks for older persons to record their feedback and adherence to the reconditioning program. Older persons were also provided with instructional materials on the reconditioning exercises (McComb et al. 2018). Ryan et al. (2022) included a webinar to introduce the topic to the expert panel, which ensured that the participants were well‐informed and prepared for their participation. The expert panel meetings and involved party consultations were conducted virtually, allowing older persons from different geographic locations to participate (Ryan et al. 2022).

## 5 Discussion

Our scoping review identified and examined the current research literature on the participation of older persons in adopting AFC in hospitals. We only found five research studies that met our inclusion criteria and described older persons' participation in AFC adoption. Participation was limited to consultation during the implementation phase and appeared minimal and superficial. The included studies did not report on older adults' participation in the embedding and integration phases of AFC models. These findings suggest that in available research, AFC models are largely adopted without the participation of older persons. This new knowledge is concerning because AFC models may be adopted without considering the alignment of these models with older persons' perceived care needs and preferences.

Older persons' participation is critical in ensuring that healthcare providers understand their priority needs and how their salient experiences influence what they consider matters in their care (Verma et al. 2017). Allowing older persons to participate in the adoption of AFC enabled healthcare organizations to tailor these models to specific contexts experienced by hospitalized older persons (Mudge et al. 2022, 2023) and address the aspects of care that are a priority to them (Glasson et al. 2006; Kelley et al. 2011). However, the participation of older persons in adopting AFC models in hospitals is poorly studied, with only five included studies examining this approach. This limited information calls for further research in this area to determine whether the participation of older persons in AFC adoption is more common in practice (and whether older persons are involved beyond the implementation phase and in varying degrees and levels of participation) than suggested by the studies identified in this review.

The majority of included studies excluded older persons with cognitive impairment, and only one study (Kelley et al. 2011) involved family caregivers as proxy informants whose perspectives are important but may differ from those of older persons (Harrison Dening et al. 2016; Wammes et al. 2021). Evidence suggests that older persons with cognitive impairment can contribute to the process of adopting AFC models. For example, Miah et al. (2020) showed that older persons with dementia and their family caregivers can actively influence the research design, study materials, and dissemination when supported through structured participation strategies. Future research should explore which inclusive approaches work best in projects that include older persons with cognitive impairment in adopting AFC, such as using plain language, conducting research activities in familiar environments, and incorporating familiar objects as visual aids (Hogger et al. 2023). Notably, family caregivers were rarely included as proxy informants to facilitate the involvement of older persons with cognitive impairment. Using participation frameworks, such as PFEF (Carman et al. 2013), that explicitly describe the role of patients and family caregivers across multiple levels and degrees of participation can help address this critical gap. The lack of participation of older persons in adopting AFC models may lead to healthcare improvements that fail to meet their perceived needs. For example, Cetin‐Sahin et al. (2022), who had only healthcare provider participants and did not include older persons in examining the adoption of an AFC model in four EDs, only identified healthcare provider‐level, organizational‐level and structural‐level factors influencing the AFC practices and missed to identify patient‐related factors. In contrast, one of the included studies, which similarly examined the adoption of the AFC model in the ED but included older persons as participants, resulted in older persons‐focused recommendations (Kelley et al. 2011). These AFC improvement recommendations in the ED included strategies to address the current issues experienced by older persons, such as reducing clutter, improving way‐finding, improving communication, and reconfiguring physical design elements to promote older persons' independence, mobility, and safety (Kelley et al. 2011). The discrepancy in identifying patient‐level factors in the results of these two studies highlights the importance of involving older persons in AFC model adoption to identify and address their perceived needs and to avoid missing their concerns when they are not involved.

Our scoping review identified a research gap that limited research studies incorporate older persons' participation in adopting AFC models in hospitals. In a systematic review, Tavares et al. (2021) comprehensively summarized different AFC models across health settings (primary, secondary, and tertiary) and identified a number of existing studies on this topic. Their findings showed that out of 34 studies included in their review, 23 studies focused on AFC models for hospital settings (Tavares et al. 2021). However, only one study (Kelley et al. 2011) met the inclusion criteria of our review. This observation supports our findings that research studies on the AFC model for hospital settings rarely incorporate the participation of older persons in their adoption process. We also want to point out that their systematic review aimed to examine the components of AFC models based on the World Health Organization's (WHO) age‐friendly guidelines (Tavares et al. 2021), which differed from the theoretical positioning of our review. However, despite the WHO (2015) guidelines highlighting the importance of older persons' participation as one aspect of creating age‐friendly environments, Tavares et al. (2021) did not explore the AFC models based on this component. The lack of attention to older persons' participation in adopting the AFC model in research could be partly explained by the limited emphasis on guidelines document, the narrow view in the scope adoption process and the importance of involving older persons, and the barriers identified in our review. More generally, this omission may reflect a broader issue of systemic ageism, where older persons' voices and contributions are undervalued and overlooked, a potential focus of future research. Future research should explore how ageism influences the limited participation of older persons in AFC adoption.

Our findings revealed that older persons' participation was limited to consultation through surveys, interviews, or written feedback. Researchers have varying perspectives on the appropriate degree of patient participation. Some authors advocate for more active patient participation, in which patients possess equal power in decision‐making in healthcare improvements (Meskó and Debronkart 2022), which was not evident in the included studies of our review. Following this perspective, patient participation becomes a form of tokenism, a gesture or an attempt to engage patients for purposes other than integrating their needs and preferences into health improvement (Majid 2020), when equal power is not achieved. However, other authors suggest that empowering older persons to participate is more important than aiming for higher degrees of participation. Dizon et al. (2019) recommend that healthcare providers and organizations explore meaningful participation for older persons when engaging them in healthcare improvements. This perspective on participation empowers older persons to determine the degree and level of their participation rather than having it defined by healthcare providers and organizations (Dizon et al. 2019), which differs from practices observed in our study. The intent, description, and manner of older persons' participation in the included studies were either poorly explained or missing and were decided by the project leads and researchers. Thus, there is a need for health organizations to prioritize understanding and recognizing meaningful participation for older persons and tailor their involvement in AFC adoption based on this understanding rather than expecting a higher degree of participation.

Included studies in our review addressed only the implementation of AFC models, the initial phase of NPT, which involved identifying problems, developing models, pilot testing, and initial evaluation of models. This finding suggested that, based on studies involving older persons, AFC model adoption remained centred on the implementation phase rather than the embedding and integration phases. This observation was consistent with earlier studies of AFC models that were focused on determining the definition and components (Boltz et al. 2010; Horgan et al. 2020; Parke et al. 2017), exploring barriers and facilitators (Stimpfel and Gilmartin 2019), and evaluating effectiveness (Khadaroo et al. 2020; Sinha et al. 2018). We anticipate that in the future, studies reporting details about how organizations and hospitals embed and integrate AFC models will emerge, similar to the quality improvement work conducted by Carney et al. (2023) in examining the adoption of AFC in a whole health system. These authors summarized the stages of their transformation to an age‐friendly healthcare system that followed the process of initiation, assessment, planning, implementation, and sustainability (Carney et al. 2023). Interestingly, similar to the findings of our scoping review, older persons' participation in this transformation process was absent (Carney et al. 2023). This observation highlights a gap in research suggesting that older persons' participation was mainly found in the implementation phase of AFC models and that future studies should incorporate older persons' participation as a focal point in the embedding and integration phases, or incorporate it in all phases of adopting AFC models.

Incorporating older persons' participation can be a challenging process. The barriers and facilitators outlined in our included studies were limited and insufficiently documented. Existing literature on patient participation cited common barriers including the resources required (Baker et al. 2016), lack of standardization in the degree and level of participation (Corrado et al. 2020), lack of organizational structure or support, poorly defined processes (Gilardi et al. 2016; Majid and Gagliardi 2019; Palumbo 2016), dominance of the biomedical model (Palumbo 2016), and diverging patient and healthcare provider perspectives (Jaensch et al. 2019; Palumbo 2016). Our findings aligned with some of these barriers and facilitators, such as time as a resource and a poorly defined process. However, we noticed the distinctive influence of patient‐related factors when older persons participated in adopting AFC models, such as their clinical status, physiological ability, presence of family caregivers, and cognitive function, and the lack of society‐related factors. These findings may suggest that patient‐related factors were assumed to significantly affect an older person's ability to participate or that researchers who conducted our included studies failed to explore society‐related factors. Although we did not find any society‐related factors, the assumptions that some older persons were denied the opportunity to participate may indicate underlying ageism in society and healthcare, which could explain the limited number of studies in our review. For example, one study attributed the low adherence to their proposed intervention to older persons' misunderstanding of the use of logbooks in recording their performance (McComb et al. 2018). Regarding the research articles captured in our search but excluded, we questioned the extent to which ageism influenced the researchers' decision not to involve older persons when adopting AFC models. Thus, to effectively address the barriers and support facilitating factors for older persons' participation in adopting AFC models, further research is needed to identify and fully understand other factors, including ageism, that may enable or mitigate their involvement.

## 6 Clinical Relevance

We are unaware of any literature synthesis exploring the research evidence available on older persons' participation in adopting AFC models in acute care hospital settings. Our study adds knowledge to existing reviews on AFC models that summarize the implementation (Gomes et al. 2022; Hogan et al. 2017; Horgan et al. 2020; Tavares et al. 2021; Wissanji et al. 2022), outcomes (Hogan et al. 2017), and spread and scale (Rogers et al. 2022) of AFC models. Our review suggests that existing AFC models' adoption did not have older persons' participation that poses a threat to aligning these models to the care preferences and perceived needs of older persons. This information is critical to healthcare professionals, researchers, and hospital organizations to ensure that older persons' perspectives and preferences are considered and integrated in healthcare improvements during AFC model adoption.

Based on our findings, we propose the following recommendations for those adopting AFC models. First, our scoping review revealed that older persons' participation in adopting AFC models remained infrequent and limited to the implementation phase despite the potential benefits of aligning these models to person‐centred principles. This finding highlights the need to increase the participation of older persons in adopting AFC models. Insights from older persons during their participation can inform the design and refinement of AFC models that integrate their perspectives, which may be typically invisible to healthcare providers and leaders (Fischer et al. 2019). To demonstrate the value and promote spread and scale, future research should investigate the impact of older persons' participation in the adoption of AFC models on patient outcomes such as safety, quality of care, quality of life, and satisfaction, implementation outcomes, and resource outcomes. While recent research focuses on identifying factors that facilitate the embedding and integration of AFC models (Burke et al. 2022), we suggest that researchers explore and incorporate older persons' participation in these phases.

Second, we recommend that healthcare providers and organizations intentionally seek meaningful participation for diverse groups of older persons. This strategy should involve an inclusive approach that considers the intersectionality of ethno‐sociocultural and person‐related factors in recruiting older person participants. Since older persons are a heterogeneous group, their intent and ability to participate might vary and could be influenced by patient‐related factors such as their energy level, clinical status, and cognitive function (Glasson et al. 2006; Kelley et al. 2011; McComb et al. 2018) as identified in our scoping review. While not addressed in our included studies, it is important to acknowledge the impact of ethno‐sociocultural factors, such as race, gender, and social status, on older persons' willingness and ability to participate (Milani et al. 2021; Principi et al. 2012) and should be considered when engaging older persons to participate in AFC adoption.

Third, hospital organizations should prioritize establishing structures and processes to enable older persons to participate in AFC adoption, especially those with cognitive impairment who are most vulnerable and often excluded from studies. Older persons' willingness and ability to participate may range from consultation level, such as getting information and having the ability to ask questions (Ekdahl et al. 2010), to partnership and shared leadership level, such as becoming active leaders in decision‐making for healthcare designs and policies (Meskó and Debronkart 2022). Involving older persons should be intentional and well‐planned out that it can be guided using frameworks. For example, McNeil et al. (2016) suggested a five‐stage framework that included components of the environment, plan, establish, build, and transition to engage older persons to participate in healthcare research and planning. Similarly, engaging older persons with cognitive impairment in research should be deliberate, using strategies to promote their participation and guided by ethical procedures that enable rather than restrict participation, such as considerations when seeking consent and assent, respecting dissent, and determining appropriate involvement of family caregivers as dyad participants (Black et al. 2010). Additionally, some authors suggest that older persons from the boomer generation will be more keen to participate in managing their health and designing healthcare services (Gill and Cameron 2022) than earlier generations (Casado et al. 2020). Thus, building capacity for older persons' participation is a timely proactive approach as more older persons enter retirement age.

## 7 Limitations

Despite conducting a comprehensive search across six health research databases, we acknowledge the limitation of a low number of included studies, which were published only in Canada and Australia. This limitation signaled the scarcity of empirical evidence on older persons' participation in adopting the AFC model in hospitals. We are cognizant of the potential publication bias from not conducting a systematic search of gray literature and excluding quality improvement reports. Within the included studies, the timing (during care at bedside (Glasson et al. 2006; Kelley et al. 2011), shortly before and after implementation (McComb et al. 2018; Mudge et al. 2022, 2023), or experience from previous hospitalization (Ryan et al. 2022)) when responding to the research varied, which may introduce recall bias. Excluding non‐research literature was our conscious decision to align our findings with our research aim of identifying research gaps. Critical appraisal of the included studies was not carried out, which is an accepted practice when conducting scoping reviews (Peters, Marnie, et al. 2020). Additionally, since four authors have gerontological research expertise, we decided to bypass the discretionary stage of expert panel consultation in our scoping review. Lastly, we recognize that we missed the opportunity to collaborate with patient partners in conducting this scoping review. McCarron et al. (2021) described a scoping review design that involved patients as a feasible and rewarding method for both patients and researchers.

## 8 Conclusion

While healthcare organizations continue to adopt AFC models in hospitals to address the challenges faced by a growing number of diverse groups of older persons, promoting their participation in the adoption process is crucial. This approach ensures that the shift from traditional care models to AFC models aligns with older persons' unique needs, values, and preferences. When seeking older persons' participation, it is essential to identify activities they can accomplish and that are meaningful to them. However, as our findings highlighted, the knowledge of older persons' participation in adopting AFC models is cursory and poorly described in the literature. This knowledge gap emphasized the need to enable older persons to participate in adopting AFC models through the phases of implementation, embedding and integration. Expanding the body of literature in this area can provide valuable knowledge on whether older persons' participation can result in the successful adoption of AFC models in hospitals and across healthcare systems.

## Author Contributions

**Kathleen F. Hunter:** conceptualization, investigation, writing – review and editing, methodology, validation, supervision, writing – original draft. **Matthias Hoben:** conceptualization, writing – original draft, writing – review and editing, methodology, validation, supervision. **Kaitlyn Tate:** validation, writing – review and editing, supervision, methodology. **Sherry Dahlke:** conceptualization, methodology, validation, writing – review and editing. **Nick Anthony Millar:** conceptualization, investigation, writing – original draft, methodology, validation, formal analysis, writing – review and editing.

## Funding

The authors have nothing to report.

## Ethics Statement

The authors have nothing to report.

## Conflicts of Interest

The authors declare no conflicts of interest.

## Supporting information


**File S1:** PRISMA Checklist.

**File S2:** Older person participation in age‐friendly care model scoping review search protocol: Ovid medline.

## Data Availability

The data that support the findings of this study are available from the corresponding author upon reasonable request.
