# Supplementary material for: The Participation of Older Persons in the Adoption of Age‐Friendly Care Models in Hospital Settings: A Scoping Review
Source: Nurs Health Sci. 2026 Jan 4;28(1):e70274. doi: 10.1111/nhs.70274 (PMC12765588; doi:10.1111/nhs.70274)
Supplement: Supplementary file 2 — File S2: Older person participation in age‐friendly care model scoping review search protocol: Ovid medline. [file NHS-28-e70274-s002.docx]

## Supplementary File 1

*Older Person Participation in Age-Friendly Care Model Scoping Review Search Protocol: Ovid Medline*

| **#** | **Query** | **Results from 6 Dec 2022** |
| --- | --- | --- |
| 1 | exp Geriatrics/ or exp Aged/ or Health Services for the Aged/ or Senior Centers/ or (elders or elderly or geriatric* or gerontolog* or "old age" or "senior citizen*" or (seniors not "high school") or ((older or mature) adj3 (adult* or person* or people or patient or patients or man or men or woman or women)) or centenarian* or nonagenarian* or octogenarian* or septuagenarian* or sexagenarian* or dottering or decrepit or tottering or overaged or "oldest old" or supercentenarian*).mp. | 3,627,820 |
| 2 | exp Hospitals/ | 310,449 |
| 3 | ("acute care*" or "inpatient*" or "critical care*" or "surgical*" or "hospital*" or "intensive care" or "patient* admit*").mp. | 3,436,778 |
| 4 | ("model*" or "framework*" or "guide*" or "approach*" or "service*" or "care*").mp. | 9,511,878 |
| 5 | ("Nurses Improving Care to Health System Elder*" or "NICHE*" or "Yale model of Care for the Elderly*" or "Acute Care for the Elderly*" or "ACE unit*" or "Age Friendly Health System*" or "4M" or "Elderly Friendly Hospital*" or "Senior Friendly Care Framework*" or "SfCare*" or "Elderly Friendly Approaches to the Surgical Environment*" or "EASE*").mp. | 119,636 |
| 6 | ("senior friend*" or "age friend*" or "elder* friend*").mp. | 819 |
| 7 | "health care (non mesh)"/ or *health services administration/ or *patient care management/ or *"delivery of health care"/ | 72,065 |
| 8 | 2 or 3 | 3,451,568 |
| 9 | 4 or 7 | 9,511,878 |
| 10 | 6 and 9 | 624 |
| 11 | 1 and 8 and 10 | 110 |
| 12 | 1 and 5 and 8 | 2,448 |
| 13 | 11 or 12 | 2,528 |
| 14 | [participation.mp](http://participation.mp/). or *Patient Participation/ or *Stakeholder Participation/ | 218,370 |
| 15 | ("participat*" or "involv*" or "feedback" or "consult*" or "engag*" or "patient engag*" or "co-design*" or "co-produc*" or "co-led*" or "co-lead*").mp. | 3,670,328 |
| 16 | 14 or 15 | 3,670,328 |
| 17 | 13 and 16 | 456 |
